# Facial Expression Recognition of Instructor Using Deep Features and Extreme Learning Machine

**DOI:** 10.1155/2021/5570870

**Published:** 2021-04-30

**Authors:** Yusra Khalid Bhatti, Afshan Jamil, Nudrat Nida, Muhammad Haroon Yousaf, Serestina Viriri, Sergio A. Velastin

**Affiliations:** ^1^Department of Computer Engineering, University of Engineering and Technology, Taxila, Pakistan; ^2^Swarm Robotics Lab, National Centre for Robotics and Automation (NCRA), Rawalpindi, Pakistan; ^3^Department of Computer Science, University of Kwazulu Natal, Durban, South Africa; ^4^School of Electronic Engineering and Computer Science, Queen Mary University of London, London E1 4NS, UK; ^5^Department of Computer Science and Engineering, Universidad Carlos III de Madrid, Leganés, Madrid 28911, Spain

## Abstract

Classroom communication involves teacher's behavior and student's responses. Extensive research has been done on the analysis of student's facial expressions, but the impact of instructor's facial expressions is yet an unexplored area of research. Facial expression recognition has the potential to predict the impact of teacher's emotions in a classroom environment. Intelligent assessment of instructor behavior during lecture delivery not only might improve the learning environment but also could save time and resources utilized in manual assessment strategies. To address the issue of manual assessment, we propose an instructor's facial expression recognition approach within a classroom using a feedforward learning model. First, the face is detected from the acquired lecture videos and key frames are selected, discarding all the redundant frames for effective high-level feature extraction. Then, deep features are extracted using multiple convolution neural networks along with parameter tuning which are then fed to a classifier. For fast learning and good generalization of the algorithm, a regularized extreme learning machine (RELM) classifier is employed which classifies five different expressions of the instructor within the classroom. Experiments are conducted on a newly created instructor's facial expression dataset in classroom environments plus three benchmark facial datasets, i.e., Cohn–Kanade, the Japanese Female Facial Expression (JAFFE) dataset, and the Facial Expression Recognition 2013 (FER2013) dataset. Furthermore, the proposed method is compared with state-of-the-art techniques, traditional classifiers, and convolutional neural models. Experimentation results indicate significant performance gain on parameters such as accuracy, *F*1-score, and recall.

## 1. Introduction

Facial expression recognition from images and videos has become very significant because of its numerous applications in the computer vision domain such as in human-computer interaction [1], augmented and virtual reality [2, 3], advance driver assistance system (ADASs) [4], and video retrieval and security systems [5–7]. Human emotions have been examined in studies with the help of acoustic and linguistic features [8], facial expressions [9–12], body posture, hand movement, direction of gaze [13], and utilization of electroencephalograms (EEGs) and electrocardiograms (ECGs) [14]. The facial expressions of an instructor impact a number of things including the learning environment, the quality of interaction in a classroom, classroom management, and more importantly the relationship with students. Instructors exhibit numerous emotions which are caused by various reasons [15], e.g., an instructor may experience joy when an educational objective is being fulfilled or when students follow given directions. When students show a lack of interest and unwillingness to grasp a concept, it causes disappointment. Similarly, anger is reflected when students lack discipline. According to teachers, these facial expressions often arise from disciplinary classroom interactions, and managing these facial expressions frequently helps them in achieving their goals [16].

Automating instructor's expression recognition can improve traditional learning and lecture delivery methods. Instructors benefit from feedback, but intensive human classroom observations are costly, and therefore, the feedback is infrequent. Usually, the received feedback focuses more on evaluating performance than improving obsolete methods [17]. One traditional solution is “student evaluation of teachers (SETs)” [18], a survey-based assessment where students mark individual teacher across various parameters on a predefined scale range. The parameters include instructor's knowledge of course content, command on lecture delivery, interaction with students, lecture material delivery, and punctuality. Such manual assessments might not be too reliable as students might only worry about their grades resulting in superficial feedback. Apart from this, the process is time-consuming and the legitimacy of acquired data is still vague [17]. Marsh [19] aims to automate instructor's feedback by using an instructor's self-recorded speech recognition while delivering lectures. This approach utilizes instructor's discourse variables and promotes student learning by providing objective feedback to instructors for improvement. In [20], a real-time student engagement system is presented which provides personalized support from instructors to those students who risk disengagement. It helps allocate instructor's time based on students who need most support as well as improving instructor's classroom practices. In [21], an intelligent tutoring system (ITS) is reported which aims to fill the gap in learning outcomes for students having diverse prior ability by incorporating real-time instructor's analytics. The introduced system, Lumilo, pairs mixed-reality smart glasses with the ITS. This creates alerts for instructors when students need help which the tutoring system is unable to provide.

Facial expression recognition (FER) methods can be categorized into two types: traditional methods and deep learning-based methods. On the basis of feature representations, a FER system can be divided into two main categories: static image-based system and dynamic sequence-based system. The static image method [22] uses spatial information from a single (current) image for encoding facial expression whereas sequence-based methods consider temporal information from adjacent frames [23]. FER methods based on traditional handcrafted features extraction can mainly have two categories of facial features: appearance-based feature extraction and geometric feature extraction. Appearance-based [24] methods describe the face texture and consider the whole face information or specific regions such as the eyes, nose, and mouth [25]. Appearance-based FER features are also extracted by applying techniques such as the Gabor wavelets transform [8], histogram of oriented gradients (HOGs) [26], local binary pattern (LBP) [27], or scale-invariant feature transform (SIFT) [28]. In [29], a Haar classifier is utilized for detecting faces followed by feature extraction using histograms of local binary patterns (LBPs). Although it is implemented in real time, it has the limitation of classifying frontal faces only. In [30], color and depth are used with a low-resolution Kinect sensor. The sensor takes input data and extracts features using the face tracking SDK (software development kit) engine, followed by classification using a random forest algorithm. However, appearance-based features are prone to errors as they can be illumination-sensitive. Geometric-based methods extract the shape of faces and estimate the localization of landmarks (e.g., eyes and nose) [31]. They detect landmarks from the face region and track facial points using an active appearance model (ASM), which is a reliable approach to address the illumination challenges faced in appearance-based methods. A study presented in [32] utilizes a Kinect sensor for emotion recognition. It tracks the face area using active appearance model (AAM). Fuzzy logic helps in observing the variation of key features in AAM. It detects emotions using its previous information from the facial action coding system. This work is limited to single subjects with only three expressions. These geometry-based and appearance-based methods have the common disadvantage of having to select a good feature to represent facial expression. The feature vector in geometry-based features is linked with landmarks, and incorrect detection of the landmark points may cause low recognition accuracy. Appearance-based features are less robust to face misalignment and background variations [33]. Incorporating these descriptors in color, gray value, texture, and statistical deformable shape features can make a robust input for the performance of architecture [34]. In general, handcrafted features are sensitive to variations in pose, aging, and appearance of the face. On the other hand, these traditional approaches require low memory as compared to neural network-based approaches. Hence, the aforementioned approaches are still utilized in the research domain for real-time embedded applications [35].

Deep learning algorithms have been applied in facial expression recognition (FER) for addressing the aforementioned issues along with different learning tasks [36]. In deep learning algorithms, the process of feature extraction uses an automatic approach to identify and extract distinct features. Deep learning algorithms comprise a layered architecture of data representation. The final layers of the networks serve as high-level feature extractors and the lower layers as low-level feature extractors [37]. Recurrent convolution networks (RCNs) [38] have been introduced for video processing. They apply convolutional neural networks on frames of videos which are then fed to a recurrent neural network (RNN) for the analysis of temporal information. These models work well when target concepts are complex with limited training data but have limitations in case of deep networks. So to overcome this issue, a model called DeXpression [39] has been devised for robust face recognition. It consists of a pair of feature extraction blocks working in parallel having layers such as convolutional, pooling, and ReLU. It uses multiple feature fusion instead of single features for achieving better performance. Another graphical model known as deep belief network (DBN) [40] was proposed. The model is based on unsupervised learning algorithms like autoencoders [41]. In [41], a hybrid RNN-CNN approach is employed for modeling the spatiotemporal information of human facial expression. They combined different modalities and performed fusion at decision level and feature level, achieving better accuracies than single modality classifiers. Similarly, in [42], a multitask global-local network (MGLN) is proposed for facial expression recognition which combines two modules: a global face module (GFM) which extracts spatial features from the frame having peak expression and a part-based module (PBM) which learns temporal features from eyes, mouth, and nose regions. Extracted features of GFM through a CNN and PBM through a long short-term memory (LSTM) network are then fused together to capture robust facial expression variation. In [43], a shallow CNN architecture is proposed with dense connectivity across pooling while dropping the fully connected layer to enforce feature sharing. Under limited training data, it achieves good performance for the effective representation of facial expressions, but the pretrained DenseNet40 and DenseNet121 show performance degradation due to overfitting. To combat the challenges of limited data in the domain of deep learning, a method presented in [33] introduces novel cropping and rotation strategies to make data abundant as well as useful for feature extraction using a simplified CNN. The cropping and rotation method removes redundant regions and retains useful facial information, and the results on CK+ and JAFFE are competitive.

The perception of information processing has completely changed by these deep learning approaches. Due to its remarkable ability of self-learning, deep learning is considered to be a better option for vision and classification problems [44]. Other approaches for classification include pretrained networks which reduce the process of long training by introducing the use of pretrained weights [45]. However, learning here involves tuning of millions of network parameters and huge labeled data for training. Since FER is significantly relevant to a number of fields, we believe FER using deep features can be applicable in understanding the semantics of instructor's facial expressions in a classroom environment.

The model proposed here aims to automate the recognition of an instructor's facial expression by incorporating visual information from lecture videos. Automatic assessment of instructors through emotion recognition may improve their teaching skills. Such an assessment mechanism can save time and resources which are currently utilized to fill up bundles of survey forms.

The main contributions of this paper are as follows:

- A novel feedforward neural network has been proposed for instructor's facial expression recognition, which is fast and robust.
- The proposed fast feedforward-based technique can learn deep neural features from any type of convolutional neural networks.
- A new dataset of instructor's facial expressions in classroom environments has been produced. Online lecture videos of instructors delivering lectures encompassing a variety of STEM (science, technology, engineering, and mathematics) disciplines have been collected.
- A new research domain was introduced by proposing a method of utilizing instructor's facial expressions in a classroom environment using computer vision techniques.

The rest of the paper is organized as follows. In Section 2, the proposed methodology for instructor's expression recognition is presented. To evaluate the proposed methodology, the experimental results are presented in Section 3.  Section 4 concludes with a summary of the achievements of the proposed method.

## 2. Proposed Methodology

**Figure 1:**
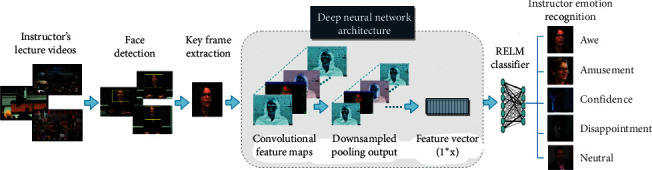
Framework of the proposed instructor's facial expression recognition.

The general framework of the proposed facial expression recognition system is presented in Figure 1. Its first step involves face detection of an instructor from the lecture videos. The extracted instructor's face frames are then subjected to key frame extraction where redundant frames are discarded and only midframes are kept for each expression. These key frames are further processed to extract deep features from different layers of a CNN. Then, these extracted deep features are used to train an RELM classifier for recognizing instructor's facial expressions within five classes. RELM is one of the variants of the extreme learning machine (ELM), which is based on a structural risk minimization principle. The structural risk minimization principle is used to optimize the structure of ELM, and regularization is utilized for accurate prediction. This principle is effective in improving the generalization of ELM. These steps are further explained in the following sections.

### 2.1. Face Detection and Key Frame Selection

**Figure 2:**
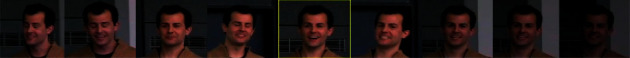
Representation of selected middle key frame for the expression of “amusement” from the shot of nine frames.

Recognizing an instructor's facial expressions is challenging because it is different from the conventional facial expression recognition system. Essentially, the data are acquired in a classroom environment. This involves challenges like face invisibility, e.g., when the instructor is writing on the board, occlusion, e.g., when the instructor is reading the slide from the laptop and half of the face is hidden behind the screen, and varying lightening conditions, e.g., when the instructors walk under the projector's light. The proposed algorithm is designed in such a way so as to overcome such challenges. Keeping in view the indoor and single object environment, faces of instructors are detected using the Viola–Jones face detection approach [46]. The detection of faces in an image by Viola–Jones algorithm sought full upright frontal faces that also reduce the nonfacial expression frames [47]. For robust practical detection, the face must be visible to the camera; hence, only frontal faces are considered. Once the face is detected, the bounding box around the face of the instructor is cropped, to form a region of interest. According to the literature, the main step in processing videos is to segment the video into temporal shots. A shot consists of a sequence of frames. Among all the frames, a key frame provides salient information of the shot. It summarizes the content of the video by removing redundant information and delivering only significant condensed information. By utilizing shot boundary detection, we select the middle frame of the shot as the key frame [48]. Generally, a human facial macroexpression lasts for about half a second to four seconds [49]. Thus, all the frames in this time span are considered. For each expression, the first and last frames usually show a neutral expression, while the middle frame gives a good expression representation of the shot. Middle frames for each expression label are therefore selected as shown in Figure 2. Using this key frame selection procedure, redundant frames are narrowed down to only the limited frames which show the peak expression. In expression representation, only those frames are selected that best characterize the emotion. Frames exhibit different levels of expressiveness. When a person expresses an emotion, a transition from a neutral to maximum expression occurs which is known as apex [50]. Training a deep learning algorithm on every single frame may negatively impact classification accuracy. Hence, for training, we select frames that contain the apex of an expression because of their strong expression content while discarding the frames where the subject is neutral for that emotion [51]. These key frames are targeted to identify five emotions: amusement, awe, confidence, disappointment, and neutral.

### 2.2. Feature Extraction Using CNN Models

After acquiring the key frames, a deep feature representation of facial expressions is generated from a 2D-CNN. These deep learning models have layered architecture that learns features at different layers (hierarchical representations of layered features). This layered architecture allows extracting high-level, medium-level, and low-level features of an instructor's face. Two types of networks are investigated: sequential network and directed acyclic graph (DAG) [52]. A serial network has layers arranged sequentially such as in AlexNet [45], which takes 227 × 227 2-dimensional input and has 8 layers. On the other hand, a DAG network has layers in the form of directed acyclic graph, with multiple layers processing in parallel for yielding efficient results. Example models of DAG are GoogleNet [53], DenseNet201 [54], ResNet50, ResNet18, ResNet101 [55], and Inceptionv3 [56] having depth of 22, 201, 50, 18, 101, and 44 layers, respectively. Although deeper layers have high-level feature representations, that does not ensure best accuracy. Instead of acquiring features from just the last layer, features are extracted from convolution, pooling, and regularization layers. We have empirically evaluated the performance of various layers of deep networks in Section 3. From DenseNet201, features are extracted using the conv4_block9_1_*bn* layer. For AlexNet, GoogleNet, Inceptionv3, and ResNet50 features are extracted from drop7, pool5_drop_7*x*7_*s*1, activation_94_relu, and avg_pool, respectively. For ResNet101 and ResNet18, we opted for pool5.

**Figure 3:**
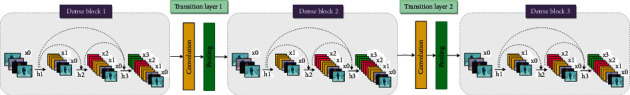
DenseNet architecture with three dense blocks and adjacent transition layers. The solid lines represent the concatenation of previous feature maps. The dashed lines show the connection of different layers.

The DenseNet architecture has been designed aiming at a maximum flow of information between layers in the network. All layers are directly connected with each other, and each layer receives feature maps produced by all preceding layers, which then transfers into succeeding layers. Unlike ResNets, here features are combined by concatenation rather than summation before passing into a layer. Hence, the *l*^th^ layer has *l* inputs, consisting of the feature maps of all preceding convolutional blocks. Its own feature maps are passed on to all *L* − 1 subsequent layers. So in an *L*-layer network, there are *L*(*L*+1)/2 direct connections unlike traditional architectures which have *L* number of connections. The *l*^th^ layer receives the featuremaps *x*^*l*^ of all preceding layers, *x*0,…, *x*^*l*−1^, which is in the following form:

(1) $$\begin{array}{c} x^{l}=H^{l}\left(\left[x^{0},x^{1},\ldots ,x^{l-1}\right]\right), \end{array}$$

where *x*^0^, *x*^1^,…, *x*^*l*−1^ are the concatenation of the feature maps in layers 0,…, *l* − 1^th^ layer. *H*^*l*^(.) is a composite function comprised of three operations which are batch normalization (BN) [57], a rectified linear unit (ReLU) [58], and a 3*∗*3 convolution (Conv). In traditional deep CNNs, layers are followed by a pooling layer that reduces feature maps size to half. Consequently, the concatenation operation used in equation (1) would be erroneous due to the change in feature maps. However, downsampling layers are an essential part of convolutional networks. To facilitate consistent downsampling, DenseNets are designed so as to divide the network into multiple densely connected dense blocks and transition layers are introduced [59].  Figure 3 shows transition layers consisting of convolution and pooling layers, present between dense blocks. The instructor feature maps are extracted from the layer conv4_block9_1_*bn* which is connected to convolution layer conv4_block9_1_conv[0][0]. Because of this dense connectivity, DenseNet requires fewer parameters as there is no need to relearn redundant feature maps, as is the case in traditional convolutional networks.

### 2.3. Classification by Regularized Extreme Learning Machine (RELM)

In this work, RELM [60] is investigated, to recognize instructor's emotions in classroom environments. The extracted 2D-CNN features of an instructor's facial emotions are fed to an RELM classifier for predicting among five emotion classes. ELM is a single hidden layer feedforward neural network (SLFN), having fast training speed and good generalization performance. However, it tends to cause overfitting as it is based on the principle of empirical risk minimization (ERM) [61]. To overcome that drawback, RELM was introduced, which is based on structural risk minimization (SRM) [62]. The ERM is based on the sum of squared error of the data samples. Having fewer data samples and small empirical risk gives less training error for training data but large testing error for unseen data, causing overfitting. Therefore, RELM works on the SRM principle which is based on the statistical learning theory. It provides the relationship between empirical risk and real risk, which is known as the bound of the generalization ability [63]. Here, the empirical risk is represented by the sum of squared error, i.e., ‖*ɛ*‖^2^ and structural risk can be represented with ‖*β*‖^2^.

Specifically, there are *n* distinct training samples (*x*_*i*_, *t*_*i*_)*ɛℝ*^*k*^*∗ℝ*^*m*^ with *g*(*x*) as the activation function. For the *i*th samples, the RELM with hidden nodes $\tilde{N}$ is modeled as

(2) $$\begin{array}{c} \sum_{i=1}^{\tilde{N}}\beta_{i}g_{i}\left(x_{q}\right)=\sum_{i=1}^{\tilde{N}}\beta_{i}g_{i}\left(w_{i}\cdot x_{q}+b_{i}\right), \end{array}$$

where *w*=[*w*_*i*1_, *w*_*i*2_,…,*w*_*m*_]^*T*^ is the weighted vector which shows the connection among hidden nodes and input nodes. *β*  =  *β*=[*β*_*i*1_, *β*_*i*2_, .., *β*_*i*_]^*T*^ represents weighted output which maintains the connection between hidden nodes to the output nodes and *b*_*i*_ is the bias of hidden layer nodes. The value *w*_*i*_ · *x*_*q*_ represents the inner product and *O*=[*o*_*j*1_, *o*_*j*2_, .., *o*_*jN*_]^*T*^ represents the output vector which is *m* × 1. So typically, if a standard SLFN with $\tilde{N}$ hidden nodes can approximate *n* distinct training samples with zero error, i.e.,

(3) $$\begin{array}{c} \sum_{i=1}^{N}\left\|o_{q}-t_{q}\right\|=0, \end{array}$$

then there must exist *w*_*i*_, *b*_*i*_ and *β*_*i*_ which satisfy the function:

(4) $$\begin{array}{c} \sum_{i=1}^{\tilde{N}}\beta_{i}g_{i}\left(x_{q}\right)=\sum_{i=1}^{\tilde{N}}\beta_{i}g_{i}\left(w_{i}\cdot x_{q}+b_{i}\right)=t_{j},\;j=1,\ldots ,N. \end{array}$$

The above equation can be written as

(5) $$\begin{array}{c} H\beta =T, \end{array}$$

where *H*(*w*_1_, *w*_2_,…, *w*_*i*_, *b*_1_, *b*_2_,…, *b*_*i*_, *x*_1_, *x*_2_,…, *x*_*i*_, )

(6) $$\begin{array}{c} =\left[\begin{array}{ccc} g\left(w_{1}\cdot x_{1}+b_{1}\right) & \ldots & g\left(w_{n}\cdot x_{1}+b_{N}\right)\\ \ldots & \ldots & \ldots \\ g\left(w_{1}\cdot x_{N}+b_{1}\right) & \ldots & g\left(w_{n}\cdot x_{N}+b_{N}\right) \end{array}\right], \end{array}$$

where *H* denotes the hidden layer output matrix of network. Traditionally, the parameters of hidden nodes are iteratively adjusted in order to reach the optimal minima. In contrast, to train an SLFN, we can arbitrarily select hidden node parameters with any nonzero activation function and can determine the output weights analytically. Hence, for finding the output matrix in reference to the theory of least squares, the estimation of *β* can be written as

(7) $$\begin{array}{c} \hat{\beta }=H^{†}T, \end{array}$$

where *H*^†^ is known as the generalized inverse of *H* also called as Moore–Penrose generalized inverse.

Here, the aim of the RELM algorithm is to find an optimum solution *β* for satisfying the following equation:

(8) $$\begin{array}{c} {\left\|H\tilde{\beta }-T\right\|}_{F}^{2}=\min_{\beta }\left({\left\|H\beta -T\right\|}_{F}^{2}\right., \end{array}$$

where ‖·‖_*F*_ is known as Frobenius norm. There are a number of regularization techniques reported in the literature such as minimax concave [64], ridge regression, and nonconvex term [65]. These terms have been utilized for linear systems to reduce the overall variance. However, when the number of hidden nodes for ELM exceeds the value 5000, it starts to overfit the model. By taking the advantage that the linear system of these SLFN's output can be calculated analytically, we use the Frobenius norm for regularization. Equation (8) can be written as follows:

(9) $$\begin{array}{c} {\left\|H\tilde{\beta }-T\right\|}_{F}^{2}=\min_{\beta }\left({\left\|H\beta -T\right\|}_{F}^{2}+\lambda {\left\|\beta \right\|}_{F}^{2}\right), \end{array}$$

(10) $$\begin{array}{c} \tilde{\beta }={\left(H^{T}H+\lambda I\right)}^{-1}H^{T}T. \end{array}$$

**Figure 4:**
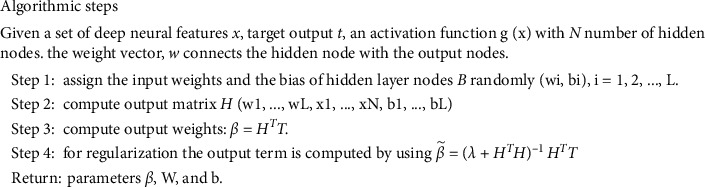
The RELM algorithm for classification.

For regularized ELM, $\tilde{\beta }$ is calculated as shown in equation (10) where *λ* is the regularization factor. When the term *λ* is a positive constant term (i.e.,  > 0), equation (10) gives the optimal solution of equation (9). By regulating *λ*, the proportion of empirical risk and structural risk can be adjusted. The optimal trade-off between these two risks will make a generalized model. The working of the proposed RELM scheme is summarized by the algorithmic steps as shown in Figure 4.

## 3. Experimentation Results and Discussion

### 3.1. Experimental Setup

For the experimentation, MATLAB 2018 has been used on an Intel Core i5 machine with 16 GB RAM and an NVIDIA TITAN XP GPU. For the evaluation of the proposed framework, four standard metrics of precision, recall, *F*1-score, and accuracy have been used. The validation schemes used for evaluating the technique are 10-fold cross-validation, leave-one-actor-out (LOAO), leave-one-sample-out (LOSO), and data split schemes (70–30%). These validation techniques define the division of training and testing sets. For example, in the 10-fold, the data are split into 10 folds where 9 folds are used to train the model and the remaining fold is used for testing and the average accuracy is recorded. The process iterates until each fold of the 10 folds has been used for testing. Similarly, for LOAO, expression samples for all actors but one are used for training. Then, testing is done using images for the unseen actor. The iteration occurs for all the actors one by one. In LOSO, all the samples for training are considered except one used as a testing sample.

### 3.2. Datasets for Facial Expression Recognition

The proposed model is evaluated on four different expression datasets, first, on a new Instructor Expression Video (IEV) dataset, created by the authors. To evaluate and explore the generalization of the proposed approach, three benchmark facial expression datasets are also explored: the Cohn–Kanade (CK) facial expression dataset [66], Japanese Female Facial Expression (JAFFE) dataset [67], and the Facial Expression Recognition 2013 (FER2013) dataset [68].

#### 3.2.1. IEV Dataset

In this new dataset, lecture videos are acquired from multiple open course-ware websites from top-ranked universities around the globe. We have incorporated the STEM (science, technology, engineering, and mathematics) domains as their applications empower students for innovative work in this technology-driven era. There are a total number of 30 actors and 30 corresponding videos with different time durations, from 20 minutes to 1 hour. To maintain some diversity, 23 are male instructors and 7 are female instructors. In each category of expressions, a total of 425 images are present, to ensure that classes are balanced. The frame resolution of each video varies from 368 × 290 to 1280 × 720 pixels with 30 frames per second. Five expression categories for instructors are identified, namely, amusement, awe, confidence, disappointment, and neutral.  Table 1 shows a tabular view of the instructor's expression dataset.

The dataset is constructed from the lecture videos of the real classroom in a realistic environment. The subjects under consideration are real-life instructors having experienced and shown emotions while delivering the session. While dealing with students, instructor's experience and express emotions from pleasure to disappointment and anger [69]. They gradually develop different schemes to regulate their genuine emotions [70]. In [69], instructors admitted to controlling the intensity of unpleasant emotions such as anger. However, the majority of the literature demonstrates the authentic display of emotions allowing instructors to display emotion in a controlled fashion [71]. In accordance with the literature and our targeted environment, we constructed a category which could be a representative of both anger and disgust. Important expressions such as anger are considered under the category of “disappointment,” as these expressions are mild in the university's classroom environment. Research [72] has shown a slight decrease in the expressiveness of instructor's unpleasant emotions such as anger, anxiety, and transforming into a form that is “disappointment and disgust”. Here, it is useful to note that disappointment frequency was five times more than the sadness frequency. In the proposed methodology, after thorough research, we intended to focus on the emotions that are conceptually salient and distinct in an academic environment. Twenty annotators including instructors and students evaluated the facial expression of instructors, and labels were assigned on the basis of majority voting.

#### 3.2.2. Cohn–Kanade

**Figure 5:**
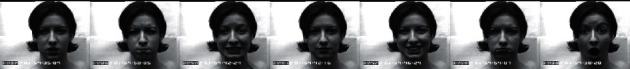
Sample sequence of seven expressions from the CK dataset.

The CK dataset [66] consists of video sequences of 97 subjects showing six expressions with a total of 582 images. Basic expressions are anger, contempt, fear, disgust, happiness, surprise, and sadness. Image resolution is 640 × 480 pixels. Sequences start with the neutral expression up to the last frame showing the emotion for that particular label. Sample images for every expression are shown in Figure 5.

#### 3.2.3. Japanese Female Facial Expression (JAFFE)

**Figure 6:**
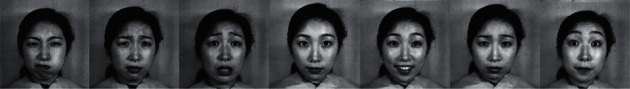
Sample images taken for each expression from the JAFFE dataset.

The JAFFE [67] database includes 213 images of 10 different female actors posing for seven facial expressions. Six of them are basic expressions: anger, joy, sadness, neutral, surprise, disgust, and fear plus one neutral as shown in Figure 6. Image resolution is 256 × 256 pixels.

#### 3.2.4. FER2013 Dataset: Facial Emotion Recognition (Kaggle)

**Figure 7:**
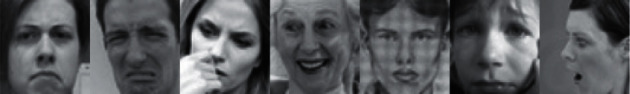
Sample images taken for each expression from the FER2013 dataset.

Facial Expression Recognition 2013 (FER2013) database [68] was introduced in the International Conference on Machine Learning (ICML) Workshop on Challenges in Representation Learning. FER2013 dataset consists of a total of 35,887 grayscale images of 48 × 48 resolution; most of them are in wild settings. It was created by using the Google image search API to scrape images through keywords that match emotion labels. FER2013 has seven emotion classes, namely, angry, disgust, fear, happy, sad, surprise, including neutral as shown in Figure 7. In contrast to the posed datasets, the challenging aspect of this dataset is variations in images, pose, partial faces, and occlusion with the use of hands.

### 3.3. Feedforward Network Tuning with Number of Nodes for Seven Convolutional Models

**Figure 8:**
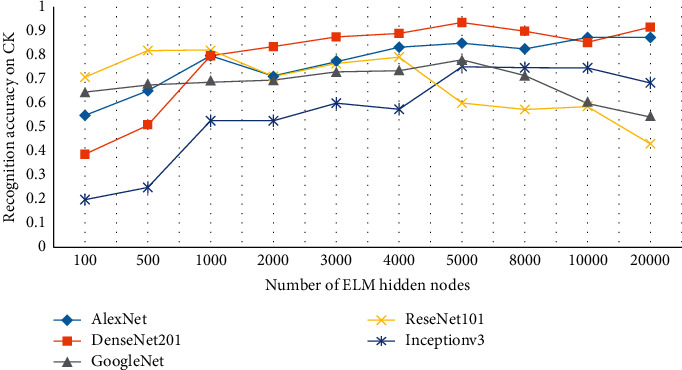
Impact of the number of nodes on expression recognition performance of the convolutional neural models on the CK dataset.

RELM assigns the initial input weights and hidden biases randomly. Through a generalized inverse operation, the output weights of SLFNs are determined analytically. However, there are parameters like the selection of the number of hidden nodes which have to be tuned to reduce classification error [73]. For tuning the hyperparameters for optimization of the proposed algorithm, a grid search approach with the range of [10–100000] has been adopted.  Figure 8 represents the hidden node behavior for five 2D-CNN networks, namely, DenseNet201, AlexNet, ReseNet101, GoogleNet, and Inceptionv3 on the CK dataset.

**Figure 9:**
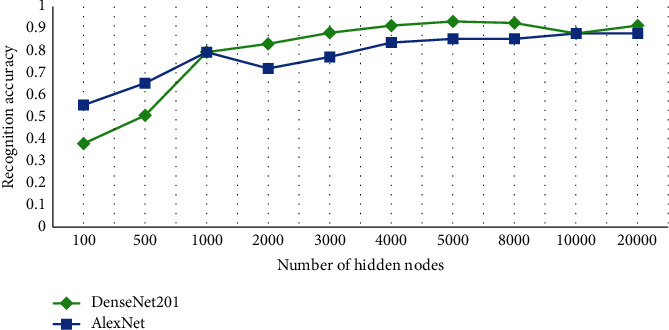
Expression recognition performance of DenseNet201 and AlexNet on the JAFFE dataset for different number of nodes.

**Figure 10:**
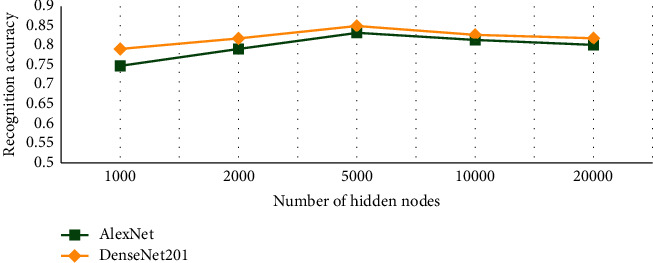
Expression recognition performance of DenseNet201 and AlexNet on the Instructor Expression Video (IEV) dataset with an increase in the number of nodes.

**Figure 11:**
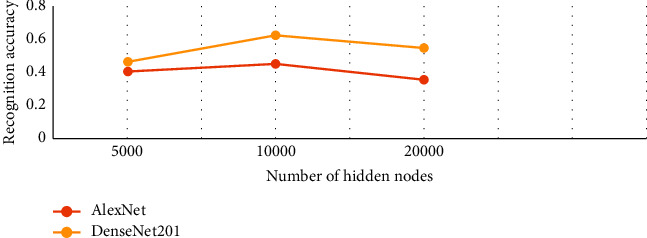
Expression recognition performance of DenseNet201 and AlexNet on the FER2013 dataset with increase in the number of nodes.

It has been observed from Figure 8 that DenseNet201 and AlexNet performed better emotion recognition among all the five deep networks. In the case of GoogleNet, we observe a consistent trend of up to 5000 nodes followed by an abrupt decrease in accuracy up to 20,000 nodes. Similarly, Inceptionv3 started from very low accuracy, increased up to 5,000, remained constant for 8000 and 10,000 nodes, and eventually showed a decrease in accuracy at 20,000. For ResNet101, initially the trend is inconsistent, followed by a steady fall after 4,000 nodes. However, DenseNet201 showed a persistent increase from 100 up to 5,000 without fluctuations. It decreases the accuracy for the next two values and showed the same amount of accuracy on 20,000 as of 5,000 nodes. A similar trend is observed in the case of AlexNet with just a slight increase at 20,000 nodes rather than a decrease. AlexNet initially showed good results in comparison with DenseNet201, but with the increase in the number of nodes, DenseNet201 outperformed AlexNet. To ensure the best model performance, this experiment has been performed to select an optimal number of nodes. On average, the optimal number of RELM nodes for all three datasets is 5000 nodes for all the five models. For Jaffe, only the top two 2D-CNN models showing better performance are selected as shown in Figure 9. A similar behavior is observed for the IEV dataset as shown in Figure 10. For the FER2013 dataset, an upward trend is observed as the number of nodes increases from 5000 to 10,000 as shown in Figure 11. In contrast to the other three datasets, FER2013 shows better performance on 10000 nodes instead of 5000. As the number of nodes increases up to 20000, the accuracy remains the same or decreases, but the training time doubles. Hence, after the empirical selection of the number of hidden nodes, these parameters will remain the same for the rest of the experiments.

#### 3.3.1. Empirical Analysis of Deep Neural Features on Standard Datasets and IEV Dataset

**Figure 12:**
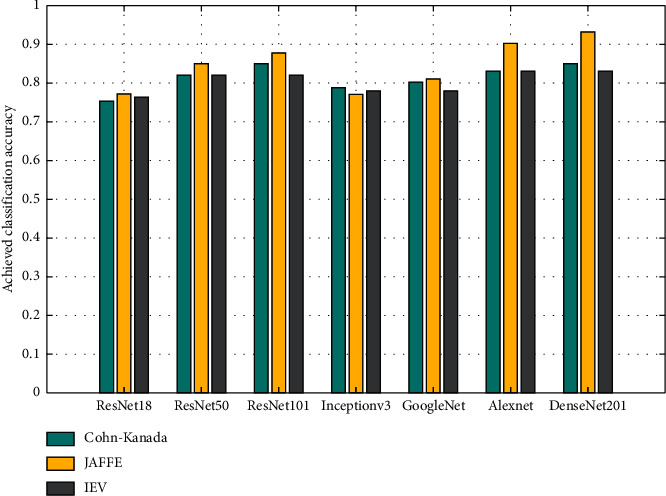
Accuracy of seven 2D-CNN models (AlexNet, DenseNet201, ReseNet18, ResNet50, ResNet100, GoogleNet, and Inceptionv3) across CK, Jaffe and IEV datasets for facial expression recognition.

Empirically, seven 2D-CNN models are evaluated here, namely, AlexNet, DenseNet201, ReseNet18, ReseNet50, ReseNet100, GoogleNet, and Inceptionv3 on two standard datasets and on the new IEV dataset. For evaluation of the proposed model, high-dimensional feature maps are extracted from all seven models, for each dataset. To visually examine the behavior, a bar chart is shown in Figure 12. It can be seen that ResNet18 gives the least accuracy among its variants followed by Inceptionv3 and GoogleNet. A possible reason for the poor accuracy of ResNet18 is the presence of an excessive number of parameters, and hence, every layer has to learn weights accordingly. In contrast, DenseNet201 outperforms other models as it has 3 times less parameters than ResNet for the same number of layers. Its narrow layers add only fewer feature maps in network collection and the classifier makes decisions accordingly.

Table 2 presents a tabular view of these models showing the statistical performance of the same experiment. Conventionally, the final fully connected layer is normally used for feature extraction, but experiments are performed on various layers and it is observed that some layers outperform the fully connected layer's accuracy. DenseNet “convolutional layer” and AlexNet “drop7” give the best accuracy of 85% and 83%, respectively. Similarly, ResNet101's pool5 layer and GoogleNet's layer “pool5_drop_7∗7_sl” give the best accuracy of 84% and 81%. Evaluation parameters for network models across corresponding layers are listed in Table 2.

Performance is evaluated on three validation schemes 10-fold, split (70–30%), LOAO, and LOSO.  Table 3 shows the results using the abovementioned layers of DenseNet201 and AlexNet on three standard datasets (Jaffe, CK, and FER2013) across all three schemes. On the Jaffe dataset, best accuracy is achieved with DenseNet201. For schemes of split (70–30%), 10-fold cross-validation, LOAO, and LOSO, the accuracies are 96.8%, 92.44%, 93.4%, and 94.55%, respectively. Similarly, results for the CK dataset are 86.59%, 80.5%, 81.59%, and 81.99% on schemes of split (70–30%), 10-fold cross-validation, LOAO, and LOSO, respectively. For the FER2013 dataset, DenseNet201 and AlexNet give accuracy of 62.74% and 45.91%, respectively.

**Figure 13:**
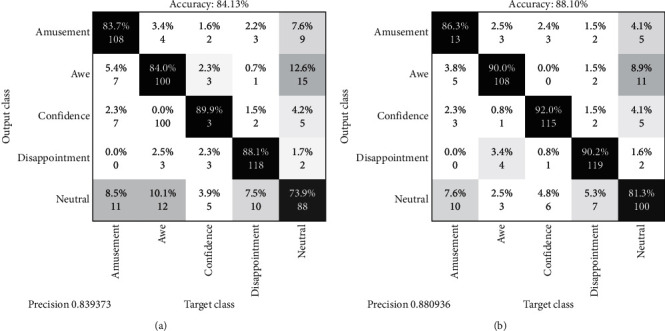
Confusion matrix of RELM and ELM on Instructor Expression Video (IEV) dataset.

Figure 13 shows the confusion matrix obtained by RELM and ELM on the IEV dataset. It shows that, with RELM, the per-class accuracy for amusement, awe, confidence, disappointment, and neutral is 86.3%, 90.8%, 92.0%, 90.2%, and 81.3%, respectively. It is validated with a 70–30% split validation scheme. Furthermore, on all four datasets, namely, CK, Jaffe, FER2013, and IEV, the total inference time taken is calculated across ELM and RELM classifiers as shown in Table 4. For Jaffe and CK, RELM outperforms ELM giving 0.7 s instead of 1.1 s and 21.8 s rather than 30.1 s, respectively. FER2013 worked best on 10000 nodes; however, the training time exceeds for RELM to 51.03 s and 17.44 s in the case of ELM. For the IEV dataset, it gives 21.28 s for ELM and 13.94 s for RELM, a difference of 7.34 s.

#### 3.3.2. Performance Comparison of Single-Layer Feedforward Network (ELM and RELM) with Traditional Classifiers

The performances of ELM and RELM classifier across traditional classifiers are empirically evaluated, as shown in Table 5. To examine the behavior, the whole framework is fixed and only the classifier in the classification part is changed. Features from five 2D-CNN models are extracted, and across every set of features, the estimated strength of ELM and RELM is tested on CK. Analysing the trend, least performance is shown by Naïve Bayes and decision tree across all the models. However, support vector machine (SVM) outperformed all the conventional classifiers. ELM and RELM show visible performance gains over all the models in comparison with ten different classifiers. ELM, contrary to the conventional backpropagation algorithm, is based on empirical risk reduction technique and needs only one iteration for its learning process. This property has made this algorithm have fast learning speed and good generalization performance yielding optimal and unique solution. For RELM, the regularization term helps in reducing overfitting without increasing computational time making a generalized instructor expression prediction model.

#### 3.3.3. Comparison with State of the Art

In this section, the performance of the proposed technique as compared to other state-of-the-art techniques on Jaffe, CK, FER 2013, and IEV datasets is compared and illustrated in Table 6. Given that it is not always possible to replicate algorithms from published results, for fair comparisons, we have used the same validation approach scheme used by each method, so findings are categorized on the basis of the validation scheme used. For JAFFE, 10-fold cross-validation results gave 92.4% accuracy outperforming the kernel-based isometric method [84], CNN method [74], and autoencoders [76] which are 81.6%, 90.37%, and 86.74%, respectively. For a 70–30 split, the proposed approach gave 96.8% whereas representational autoencoders [85] and CNN [77] lag behind. A similar situation occurs for the other two schemes as well. However, for the CK dataset, 82% is achieved for 10-fold cross-validation whereas DTAN and DTGN [75] outperform the method here with 91.4%. Similar results are observed for other validation schemes. We found the reason behind less accuracy on CK is low variance among the facial expressions and low-resolution grayscale images. In the case of FER-2013, the literature shows an overall low trend in accuracy because of high variance and occlusion conditions of the dataset. Wang et al. [79] performed multiple methods in which HOG with C4.5 classifier gave 46.1% and CNN with decision tree gave 58.8% accuracy. From exploring CNN [80] to AlexNet [82] and then to CNN ensemble [83], an increasing trend from 57.1%, 61.0%, and 62.4% is observed, respectively. In [81], VGG is incorporated with face recognition models trained on large dataset and audio features give 60% accuracy. The proposed method outperformed other state-of-the-art methods on Jaffe. This feedforward approach combined with strong classifier forms a generalized feedforward neural model for instructor expression recognition.

#### 3.3.4. Comparison with Pretrained Convolutional Approaches

Table 7 presents a comparison of accuracy along with execution times taken by the pretrained convolutional neural models on three standard datasets: Jaffe, CK, and FER2013. CNN models AlexNet and VGG16 [86] gave 93% and 96% on Jaffe, 90.2% and 92.4% on CK, and 61.1% and 59.6% on FER2013, respectively, with an execution time of 0.94 s. Similarly, ResNet101 shows 90% on Jaffe and 49% on FER2013, and Inceptionv3 [90] gives 75.8% on Jaffe and 76.5% on CK. Lastly, it is compared with the proposed model which gives 96.8% and 86.59% accuracy on Jaffe and CK, respectively, with 0.74 s average execution time taken for each emotion per frame. The proposed model shows results in 0.2% less time duration on Jaffe. These pretrained models work on backpropagation approaches where the weights are updated after every iteration. In contrast, the proposed feedforward model decreases the computational time, making it fast to learn and classify the instructor's expressions in classroom.

Our algorithm not only performs well on the annotated datasets but also demonstrates the implementation on the real-time video stream generated through webcam or any other source by framewise traversing. The face detection and key frame extraction blocks in the proposed framework clearly indicate how to handle the real-time video data to be used for facial expression in subsequent stages. For real-time results and to run the trained model on devices with low-computational strength such as mobile phones, edge devices, or embedded architectures, TensorFlow Lite may be used. To implement in a real-time environment captured through a webcam or smartphone, we only need to run an inference on embedded devices or raspberry pi with an accelerator. At inference level, testing is done frame by frame and an inference graph will be generated having confidences which could then be burned on the device as per requirement. In essence, these utilities provide full-fledged feasibility for deploying the proposed application in a real-time resource-constrained environment.

## 4. Conclusion

In this paper, a novel approach has been proposed for facial expression recognition of instructors in a classroom environment by incorporating a feedforward learning model with deep features. In contrast to backpropagation approaches, the proposed model works in a feedforward fashion. It extracts the deep features from a neural model for high-level representation gain, without updating the weights iteratively, causing a reduction in computational time complexity. Extensive experimentations are performed with state-of-the-art techniques, traditional classifiers, and other deep neural models. The proposed method has proven to be successful in evaluating five instructor's expressions in a classroom environment. For future research, we will investigate the performance of the model with more features such as instructor's speech and activity recognition approaches in order to improve the effectiveness of classroom teaching methods.

## Figures and Tables

**Table 1:** Tabular view of the new Instructor Expression Video (IEV) dataset along with sample images for each expression class.

Sample images from lecture videos	Expression	No. of samples	Gender
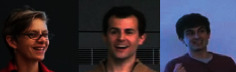	Amusement	425	Male: 23 Female: 7
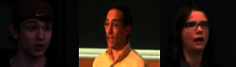	Awe	425	
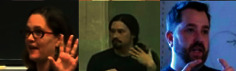	Confidence	425	
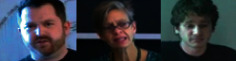	Disappointment	425	
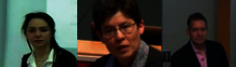	Neutral	425	

**Table 2:** Statistical performance of seven 2D-CNN models (AlexNet, DenseNet201, ReseNet18, ResNet50, ResNet100, GoogleNet, and Inceptionv3) on the Instructor Expression Video (IEV).

Network	Layer	Accuracy	Neurons	Precision	Recall	*F*1-score	Error
DenseNet201	conv4_block9_1_bn	0.85	5000	0.85	0.87	0.85	0.14
AlexNet	drop7	0.83	5000	0.83	0.85	0.83	0.12
GoogleNet	pool5-drop_7 × 7_s1	0.81	5000	0.81	0.83	0.81	0.18
Inceptionv3	activation_94_relu	0.78	5000	0.75	0.79	0.76	0.22
ResNet101	pool5	0.84	5000	0.83	0.85	0.83	0.16
ResNet18	pool5	0.75	5000	0.79	0.81	0.79	0.2
ResNet50	avg_pool	0.82	5000	0.82	0.82	0.82	0.82

**Table 3:** Results of deep model layers on standard datasets (CK, Jaffe, and FER2013) using validation schemes across RELM classifier.

Dataset	Network	Layer	Split (70–30%)	10-fold	LOAO	LOSO
Jaffe	DenseNet201	conv4_block9_1_bn	96.8	92.44	93.4	94.55
	AlexNet	drop7	91.67	86.03	84.01	85.91

CK	DenseNet201	conv4_block9_1_bn	86.59	80.5	81.59	81.99
	AlexNet	drop7	81.67	86.03	84.01	85.91

FER2013	DenseNet201	conv4_block9_1_bn	62.74	—	—	—
	AlexNet	drop7	45.91	—	—	—

**Table 4:** Execution time taken (s) for CK, Jaffe, FER2013, and IEV across both classifiers.

Database	Training time (s)		Testing time (s)	
	ELM	RELM	ELM	RELM
Jaffe	1.11	0.749	0.06	0.02
Cohn–Kanade	30.17	21.83	0.48	0.27
FER2013	17.44	51.03	0.21	0.37
IEV	13.94	21.28	0.28	0.17

**Table 5:** Comparison of ELM/RELM with the traditional classifiers on the CK dataset.

Classifiers	AlexNet			DenseNet			GoogleNet			Inceptionv3			ResNet101		
	Accuracy	Precision	*F*1-score	Accuracy	Precision	*F*1-score	Accuracy	Precision	*F*1-score	Accuracy	Precision	*F*1-score	Accuracy	Precision	*F*1-score
RELM	0.82	0.80	0.83	0.83	0.85	0.82	0.78	0.75	0.78	0.78	0.69	0.74	0.82	0.84	0.82
ELM	0.83	0.79	0.82	0.73	0.75	0.74	0.72	0.76	0.70	0.78	0.79	0.79	0.56	0.46	0.52
SVM	0.80	0.80	0.81	0.77	0.75	0.77	0.80	0.80	0.80	0.77	0.75	0.77	0.82	0.82	0.80
Naïve Bayes	0.23	0.25	0.25	0.20	0.28	0.25	0.29	0.28	0.28	0.24	0.25	0.24	0.43	0.45	0.45
Random forest	0.45	0.49	0.46	0.76	0.79	0.75	0.53	0.48	0.52	0.52	0.53	0.52	0.70	0.77	0.75
K-nearest neighbors	0.57	0.59	0.56	0.60	0.68	0.69	0.60	0.60	0.61	0.53	0.54	0.59	0.79	0.79	0.77
Logistic regression	0.29	0.31	0.29	0.53	0.52	0.52	0.55	0.59	0.56	0.58	0.60	0.59	0.59	0.60	0.61
Random tree	0.26	0.28	0.25	0.53	0.55	0.50	0.75	0.75	0.73	0.57	0.57	0.57	0.55	0.53	0.57
Simple logistic	0.48	0.47	0.48	0.73	0.74	0.70	0.73	0.75	0.75	0.75	0.78	0.75	0.74	0.74	0.74
Decision table	0.23	0.20	0.23	0.48	0.45	0.46	0.74	0.70	0.74	0.43	0.45	0.40	0.42	0.42	0.42
Multiclass classifier	0.57	0.62	0.60	0.65	0.67	0.67	0.68	0.74	0.70	0.74	0.74	0.74	0.70	0.74	0.70
Multilayer perceptron	0.43	0.43	0.41	0.65	0.70	0.69	0.78	0.68	0.67	0.68	0.65	0.66	0.65	0.68	0.68

**Table 6:** Accuracy comparison between state-of-the-art approaches on JAFFE, CK, and FER2013.

Dataset	Validation scheme	Methods	Accuracy (%)
JAFEE	10-fold	KDIsomap [74]	81.6
		EDL [75]	90.3
		CBIN [76]	86.7
		Proposed approach	**92.4**
	Split (70–30%)	RAU's [46]	86.3
		CNN [77]	76.5
		Proposed approach	**96.8**

CK	10-fold	DTAN + DTGN [31]	**91.4**
		DNN [22]	90.9
		Proposed approach	82.8
	Split (70–30%)	DCNN as SCAE [78]	**92.5**
		Proposed approach	86.5

FER2013	Split (70–30%)	HOG + C4.5 [79]	46.1
		CNN [80]	57.1
		CNN + decision tree [79]	58.8
		VGG-Face + FR-Net-A + B + C + Audio [81]	60.0
		AlexNet [82]	61.0
		CNN ensemble [83]	62.4
		Proposed approach	**62.7**

**Table 7:** Accuracy comparison between convolution neural networks on standard datasets along with time taken per frame.

CNN model	JAFFE (%)	CK (%)	FER2013 (%)	Time taken per frame (sec)	Number of parameters (million)
AlexNet [86]	93	90.2	61.1	—	61
VGG16 [86]	96	92.4	59.6	0.94	138
VGG19 [87]	93	93	60	—	144
ResNet101 [88]	90	—	49	—	44.5
Inceptionv3 [89]	75.8	76.5	—	—	23
Proposed method	**96.8**	**86.5**	62.5	**0.74**	0.005

## Data Availability

The data used to support the findings of this study are available from the corresponding author upon request.

## Abbreviations

FER: Facial expression recognition
RELM: Regularized extreme learning machine
IEV: Instructor Expression Video
CNN: Convolution neural network
SLFN: Single hidden layer feedforward neural network
LOSO: Leave one sample out
LOAO: Leave one actor out.
